# A cooperative biphasic MoO_*x*_–MoP_*x*_ promoter enables a fast-charging lithium-ion battery

**DOI:** 10.1038/s41467-020-20297-8

**Published:** 2021-01-04

**Authors:** Sang-Min Lee, Junyoung Kim, Janghyuk Moon, Kyu-Nam Jung, Jong Hwa Kim, Gum-Jae Park, Jeong-Hee Choi, Dong Young Rhee, Jeom-Soo Kim, Jong-Won Lee, Min-Sik Park

**Affiliations:** 1 Battery Research Center, Korea Electrotechnology Research Institute, 12 Bulmosan-ro 10 beon-gil, Changwon, 51543 Republic of Korea; 2 Department of Advanced Materials Engineering for Information and Electronics, Kyung Hee University, 1732 Deogyeong-daero, Giheung-gu, Yongin, 17104 Republic of Korea; 3 School of Energy System Engineering, Chung-Ang University, 84 Heukseok-ro, Dongjak-gu, Seoul, 06974 Republic of Korea; 4 New and Renewable Energy Institute, Korea Institute of Energy Research, 152 Gajeong-ro, Yuseong-gu, Daejeon, 34129 Republic of Korea; 5 Department of Chemical Engineering (BK21 FOUR), Dong-A University, 37 Nakdong-daero, Saha-gu, Busan, 49315 Republic of Korea; 6 Department of Energy Science and Engineering, Daegu Gyeongbuk Institute of Science and Technology (DGIST), 333 Techno Jungang-daero, Hyeonpung-eup, Dalseong-gun, Daegu, 42988 Republic of Korea

**Keywords:** Density functional theory, Batteries, Synthesis and processing

## Abstract

The realisation of fast-charging lithium-ion batteries with long cycle lifetimes is hindered by the uncontrollable plating of metallic Li on the graphite anode during high-rate charging. Here we report that surface engineering of graphite with a cooperative biphasic MoO_*x*_–MoP_*x*_ promoter improves the charging rate and suppresses Li plating without compromising energy density. We design and synthesise MoO_*x*_–MoP_*x*_/graphite via controllable and scalable surface engineering, i.e., the deposition of a MoO_*x*_ nanolayer on the graphite surface, followed by vapour-induced partial phase transformation of MoO_*x*_ to MoP_*x*_. A variety of analytical studies combined with thermodynamic calculations demonstrate that MoO_*x*_ effectively mitigates the formation of resistive films on the graphite surface, while MoP_*x*_ hosts Li^+^ at relatively high potentials via a fast intercalation reaction and plays a dominant role in lowering the Li^+^ adsorption energy. The MoO_*x*_–MoP_*x*_/graphite anode exhibits a fast-charging capability (<10 min charging for 80% of the capacity) and stable cycling performance without any signs of Li plating over 300 cycles when coupled with a LiNi_0.6_Co_0.2_Mn_0.2_O_2_ cathode. Thus, the developed approach paves the way to the design of advanced anode materials for fast-charging Li-ion batteries.

## Abstract (web summary)

Fast-charging of lithium-ion batteries is hindered by the uncontrollable plating of metallic Li on the graphite anode during cycling. Here, the authors demonstrate the fast chargeability and long cycle lifetimes via surface engineering of graphite with a cooperative biphasic MoO_*x*_–MoP_*x*_ promoter.

## Introduction

The demand for high-performance lithium-ion batteries (LIBs) is steadily increasing with the growth of the electric vehicle (EV) market. The success of EV implementation largely relies on LIB performance, which can be characterised in terms of energy density, safety, cycle lifetime, cost, and charging time^1–4^. In particular, charging time is regarded as a critical factor influencing customer willingness to adopt EVs, e.g., the United States Advanced Battery Consortium (USABC) intends to realise batteries that can be charged to >80% of full capacity in 15 min^5–7^. The charging performance of LIBs is determined not only by charging protocol and cell configuration but also by the choice of employed materials, particularly by that of the anode material^8^. Graphite, employed as the anode material of current LIBs, suffers from sluggish interfacial kinetics, which results in an anode voltage drop to below 0 V vs. Li/Li^+^ under fast-charging conditions and hence causes undesirable plating of metallic Li on the graphite surface^9–11^. In addition to inducing a significant capacity loss during high-rate cycling, dendritic Li plating can cause short-circuiting and thus result in LIB ignition or even explosion.

To resolve these technical issues, various nanostructured materials with tailored morphologies (e.g., nanoparticle networks^12,13^, heterogeneous nanolayers^14,15^, and porous nanoarchitectures^16^) have been intensively explored as fast-chargeable LIB anodes, and the large surface areas and short diffusion lengths of such materials have been shown to allow remarkable rate capability improvements^17^. However, the low densities of highly porous nanoscale materials result in a significant loss of LIB energy density. With this in mind, Griffith et al. proposed non-nanoscale-engineered complex Nb–W oxides (Nb_16_W_5_O_55_ and Nb_18_W_16_O_93_) as LIB anode materials, showing that they exhibit an exceptionally high-rate performance^18^. These complex oxides, comprising micron-sized particles, were found to intercalate large amounts of Li^+^ at high rates. The high operating voltages of these materials (1.0–2.5 V vs. Li/Li^+^) may require the use of high-voltage cathodes and/or rational electrode/cell designs. Given the fact that any material development requires rigorous engineering considerations for electrodes and cells, the modification of graphite-based anodes with functional materials appears to be a practical approach to realising fast-charging LIBs without sacrificing their energy density. Recently, Kim et al. reported an enhanced-rate-capability hybrid anode, composed of a nanolayer of implanted amorphous Si and edge-site-activated graphite, suggesting that this nanolayer allows fast Li diffusion into the graphite core and minimises the energy density loss of the composite material^19^. Although the corresponding LIB featured an energy density comparable to that of conventional graphite-anode LIBs, its practical applicability should be further addressed in terms of the dimensional stability of Si during cycling and the production cost.

Herein, we show that surface engineering of graphite with a cooperative biphasic MoO_*x*_–MoP_*x*_ promoter improves the fast-charging capability and long-term cycling stability of LIBs without compromising their energy density. For simplicity, the promoter will hereafter be referred to as Mo-CP, where Mo represents MoO_*x*_–MoP_*x*_, and CP stands for cooperative promoter. The Mo-CP is composed of MoO_*x*_ and MoP_*x*_, each of which serves a unique function to suppress Li plating upon high-rate charging: MoO_*x*_ plays a role in mitigating the growth of resistive films on the graphite surface, while nanoscale MoP_*x*_ hosts a large amount of Li^+^ without significant volume changes and reduces the Li^+^ adsorption energy to facilitate the interfacial kinetics of Li^+^ intercalation. As a result, a full cell assembled with a LiNi_0.6_Co_0.2_Mn_0.2_O_2_ (NCM) cathode and a Mo-CP/graphite anode is shown to exhibit exceptionally fast chargeability (<10 min charging for 80% of the capacity) and stable cycling performance over 300 cycles under fast-charging conditions.

## Results

### Surface engineering of graphite with Mo-CP

**Fig. 1:**
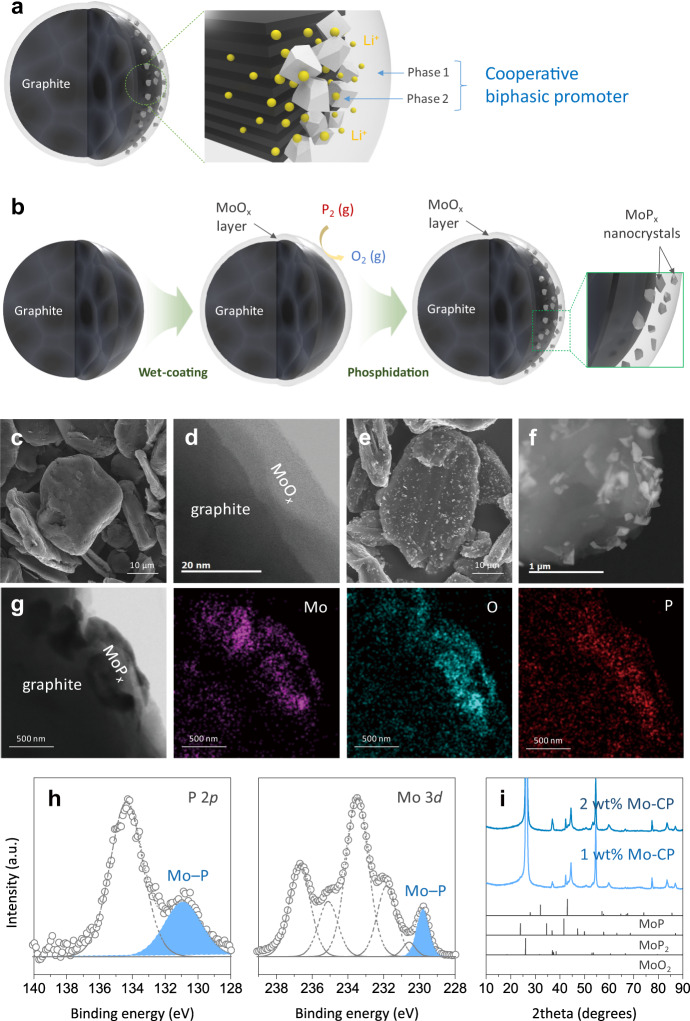
Principle of cooperative promoter design and synthesis of Mo-CP/graphite. **a** Schematic diagram of a CP/graphite anode. The CP should (1) facilitate the formation of less resistive films; (2) store Li^+^ at high potentials; (3) exhibit small volume variation; and (4) possess high affinity to Li^+^. **b** Schematic synthesis of Mo-CP/graphite via vapour-induced phase transformation of MoO_*x*_ to MoP_*x*_. **c**–**f** Field emission scanning electron microscopy (FESEM) and transmission electron microscopy (TEM) images of graphite particles obtained after MoO_*x*_ coating (**c**, **d**) and phosphidation (**e**, **f**). **g**–**i** TEM image and EDS elemental mappings (**g**), XPS spectra (**h**), and XRD patterns (**i**) of Mo-CP/graphite.

Figure 1a illustrates the concept of a fast-chargeable graphite anode modified with a functional promoter that facilitates Li^+^ intercalation into graphite while suppressing unwanted Li plating during high-current charging^20,21^. Herein, the promoter was designed based on the following principles. First, the promoter should inhibit the formation of highly resistive species, e.g., Li_2_CO_3_, which is commonly observed on bare graphite after the first several cycles. Second, it should be able to host a large amount of Li^+^ at higher voltages than graphite does, thereby preventing the anode voltage from decreasing to below 0 V vs. Li/Li^+^ at the early stage of fast charging. Third, it should not suffer from large volume changes, maintaining structural integrity over the course of cycling. Finally, considering the fact that the desolvation process of Li^+^ cations would be the rate-determining step for their intercalation into graphite^22,23^, the promoter should have a high affinity to these cations to facilitate the stripping of the solvation sheath and quickly adsorb “naked” Li^+^. The four above-mentioned principles led us to explore carbon-free, inorganic molybdenum compounds (oxides and phosphides), which are currently receiving increased attention because of their electrochemical multi-functionality^24,25^. In particular, MoP_*x*_ has been reported to exhibit remarkable catalytic properties for electrochemical reactions (e.g., hydrogen evolution)^26^, and/or high Li storage capacity^27–29^. Inspired by this, we proposed a biphasic nanolayer composed of MoO_*x*_ and MoP_*x*_, as a suitable candidate fulfilling the requirements for a cooperative promoter.

To address the fast chargeability with a minimal loss of energy density, we developed a Mo-CP/graphite anode via controllable and scalable surface engineering (Fig. 1b). As will be shown below, the proposed process allowed one to achieve a uniform deposition of Mo-CP at a low loading (1–2 wt%) on the graphite surface. A nanolayer of MoO_*x*_ (thickness ≈ 10 nm) (Fig. 1c, d) was first deposited on the graphite surface through simple wet-coating using a solution of MoO_3_ dissolved in H_2_O_2_. The resulting powder was treated with P-containing gas at 600 °C to induce the partial phase transformation (phosphidation) of MoO_*x*_ to MoP_*x*_, which resulted in the formation of abundant MoP_*x*_ nanocrystals in the MoO_*x*_ matrix (Fig. 1e, f). Energy-dispersive X-ray spectroscopy (EDS) (Fig. 1g) and X-ray photoelectron spectroscopy (XPS) analyses (Fig. 1h, Supplementary Fig. 1) confirmed the transformation of MoO_*x*_ to MoP_*x*_ and the presence of residual MoO_*x*_ on the surface of graphite. Unlike pristine graphite and MoO_*x*_/graphite, the XPS P 2*p* spectrum of Mo-CP/graphite exhibited an additional characteristic peak for MoP_*x*_ at a binding energy of 130.8 eV (Fig. 1h). The XPS Mo 3*d* spectrum indicated that Mo mainly existed with an oxidation state of Mo^6+^ at the surface of MoO_*x*_/graphite, but a fraction of Mo^4+^ in Mo-CP/graphite was increased as a result of the formation of MoP_*x*_ during the phosphidation process (Fig. 1h, Supplementary Fig. 1). The powder X-ray diffraction (XRD) patterns of Mo-CP/graphite featured characteristic peaks of MoP and MoP_2_ phases at 43.0° and 41.5°, respectively, and a peak of residual MoO_2_ at 37.3° (Fig. 1i). Given the simplicity of the wet-coating and mild heat-treatment as well as the low loading of Mo-CP (1–2 wt%), the proposed process may be implemented to modify the graphite surface at minimal material/processing cost: thus, it is easy to scale up for commercial production.

### Functionality of Mo-CP

**Fig. 2:**
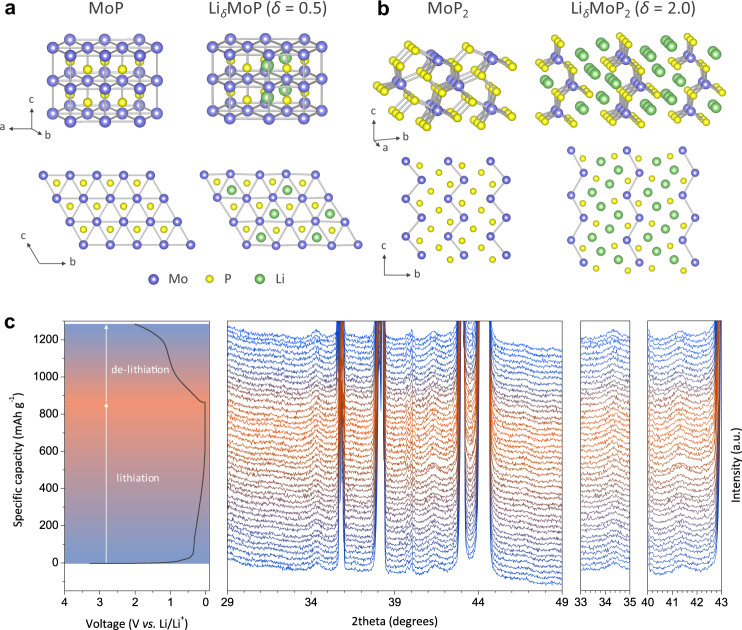
MoP_*x*_ lithiation. **a**, **b** DFT calculation-determined crystal structures and atomic arrangements of MoP/Li_*δ*_MoP (*δ* = 0.5) (**a**) and MoP_2_/Li_*δ*_MoP_2_ (*δ* = 2) (**b**). **c** Voltage profile of MoP_*x*_ and in situ XRD patterns recorded during initial lithiation/delithiation at 100 mA g^−1^ in a voltage range of 0.01–1.5 V vs. Li/Li^+^.

The functionality of Mo-CP was investigated by using both theoretical and experimental tools. For density functional theory (DFT) calculations on MoP_*x*_ (Supplementary Note 1), we carefully developed fully relaxed structures of hexagonal MoP (Fig. 2a) and orthorhombic MoP_2_ (Fig. 2b)^30^. In hexagonal MoP with a tungsten carbide-type structure, each Mo atom is surrounded by six trigonal-prismatically coordinated P atoms (Fig. 2a), and favourable Li intercalating sites without structural distortion exist between P–P bonds. A maximum of four Li atoms (*δ* = 0.5) can be intercalated into Li_*δ*_MoP with eight candidate sites, which leads to lattice expansion in the *b*- and *c*-axis directions (Fig. 2a, Supplementary Tables 1 and 2). Interestingly, the *a* lattice parameter did not change during lithiation because of the facet sharing of the Mo–P local structure. The volume expansion calculated for lithiation to Li_0.5_MoP was estimated as only 30% (Supplementary Fig. 2), which agreed with the results of TEM analysis (Supplementary Fig. 3). The breakage of the weak residual P pair resulted in the generation of interlayer space for intercalated Li. The structure of Li_*δ*_MoP_2_ was also generated by Li intercalation into the basic structure of MoP_2_ (Fig. 2b, Supplementary Fig. 4, Supplementary Tables 3 and 4). During lithiation, the interstitial Li could be positioned at the five-ring-pore by cleavage of the bond between residual P atoms, which promoted the formation of hexahedral local Mo–P structures where the central Mo atom could be coordinated by seven neighbouring P atoms. Moreover, our calculations revealed that Li_*δ*_MoP_2_ has a higher Li storage capability than Li_*δ*_MoP and experiences a volume expansion of ~66% at *δ* = 2.

For experimental verification, MoP_*x*_ particles were synthesised by a solid-state method (Supplementary Note 2). Mo and red-P precursors were mechanically milled to obtain MoP_*x*_ crystallites interconnected on the nanoscale (Supplementary Fig. 5). The *x* value of MoP_*x*_ was estimated as ~1.6 from the results of XRD analysis using the reference intensity ratio procedure^31^. To confirm theoretical predictions, we investigated the electrochemical lithiation behaviour and dimensional stability of MoP_*x*_ by in situ XRD. Figure 2c shows the in situ XRD patterns of MoP_*x*_ and its first-cycle voltage profile, demonstrating the presence of characteristic MoP_2_ (2*θ* ≈ 34.5° and 41.5°) and MoP (2*θ* ≈ 43.0°) peaks and revealing that MoP_*x*_ delivered a high Li storage capacity in the voltage range of 0.01–1.5 V vs. Li/Li^+^ (Supplementary Note 3). No significant peak shift was observed during cycling, i.e., the volume variation of MoP_*x*_ nanoparticles was small, as predicted by DFT calculations. Furthermore, the thickness change of the MoP_*x*_ electrode after lithiation was less than 25% (Supplementary Fig. 7) and comparable to that of pristine graphite^32^.

**Fig. 3:**
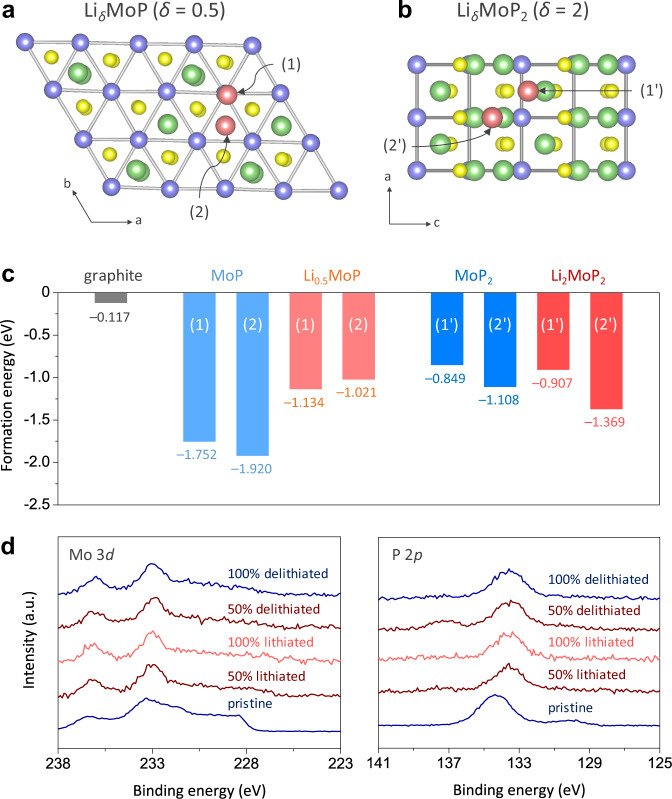
Li^+^ adsorption on MoP_*x*_. **a**–**c** Atomic arrangements (**a**, **b**) and formation energies (**c**) for Li^+^ adsorption on Li_*δ*_MoP (*δ* = 0.5) and Li_*δ*_MoP_2_ (*δ* = 2) determined by the DFT calculations. **d** Ex situ XPS Mo 3*d* and P 2*p* spectra of MoP_*x*_ recorded at different lithiation states. Lithiation and delithiation experiments were conducted by applying a constant current density of 100 mA g^−1^ in a voltage range of 0.01–1.5 V vs. Li/Li^+^.

We also conducted DFT calculations to investigate Li^+^ adsorption on Li_*δ*_MoP (*δ* = 0.5) and Li_*δ*_MoP_2_ (*δ* = 2) structures. Figure 3a, b presents Li^+^ adsorption behaviours on the [001] surface of Li_0.5_MoP and the [010] surface of Li_2_MoP_2_, respectively, demonstrating that the exposed P atoms on the [001] facet of MoP strongly bound Li, depending on the atomic environment at the underlying layer. The formation energies were calculated as –1.752 eV for site (1) and –1.920 eV for site (2) and were much lower than that of graphite (–0.117 eV) (Fig. 3c). Upon lithiation to Li_0.5_MoP, the formation energies increased to –1.134 eV at site (1) and –1.021 eV at site (2), since the existing Li–P bonds interfered with further Li–P bonding. In the case of MoP_2_, Li^+^ adsorption occurred on the facets of residual P and heptahedra (octahedra at Li_2_MoP_2_) with formation energies of –0.849 and –1.108 eV calculated for sites (1′) and (2′), respectively. The energies of Li^+^ adsorption on Li_2_MoP_2_ were slightly lower than those obtained for MoP_2_, as shown in Fig. 3c. Thus, the results of DFT calculations revealed that both MoP and MoP_2_ have a higher affinity to Li^+^ than graphite and hence promote Li^+^ adsorption during lithiation. Ex situ XPS analysis (Fig. 3d) showed that the Mo 3*d* peak at 233.7 eV shifted to a lower binding energy at the initial stage of lithiation, which was indicative of Li^+^ adsorption on the Mo–P local structure. A more evident peak shift (from 134.2 to 133.6 eV) was observed in ex situ P 2*p* spectra, indicating the breakage of P–P bonds due to Li^+^ adsorption.

The XPS analysis was also performed on pristine graphite, MoO_*x*_/graphite, and Mo-CP/graphite after the formation cycles (Supplementary Note 4). The XPS Li 1 *s* spectrum of pristine graphite exhibited a relatively large peak for Li_2_CO_3_ at a binding energy of 55.5 eV (Supplementary Fig. 8). On the other hand, the formation of resistive Li_2_CO_3_ species was effectively reduced on MoO_*x*_/graphite and Mo-CP/graphite because the direct exposure of carbon surface could be minimised by the surface modification with the conformal MoO_*x*_ coating. Thus, DFT calculations and material characterisations confirmed the functionality of Mo-CP, revealing that (i) MoO_*x*_ mitigates the significant growth of resistive films on the graphite surface; (ii) MoP_*x*_ hosts a large amount of Li^+^ without significant volume changes; and (iii) it reduces the Li^+^ adsorption energy to facilitate the interfacial kinetics of Li^+^ intercalation.

### Electrochemical performance of Mo-CP/graphite

**Fig. 4:**
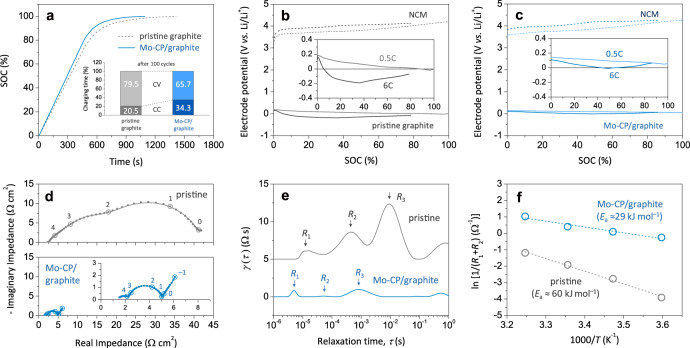
Fast-charging capability of Mo-CP/graphite. **a** SOC vs. time profiles of full cells with pristine graphite and Mo-CP/graphite anodes. Relative contributions of CC charging and CV charging modes to the total charging time after 100 cycles are presented in (**a**). **b**, **c** Voltage profiles of electrodes in full cells with pristine graphite (**b**) and Mo-CP/graphite (**c**) anodes. **d**, **e** AC-impedance spectra (**d**) and DRT plots (**e**) of half-cells. In the three-electrode half-cell, pristine graphite or Mo-CP/graphite was used as the working electrode, while Li was employed as counter and reference electrodes. Solid lines in (**d**) represent the results of fitting based on DRT analysis, and numbers above open circles in (**d**) indicate logarithmic frequency values. **f** *E*_a_ values for interfacial desolvation/migration reactions (*R*_1_ + *R*_2_) calculated from the temperature dependence of impedance.

To prove the efficacy of Mo-CP in a practical LIB, Mo-CP/graphite anodes (areal capacity = 2.2 mAh cm^–2^ and porosity = 35.0% (Supplementary Fig. 9)) containing 2 wt% Mo-CP were coupled with commercially available NCM cathodes. Full cells were designed with an *N*/*P* ratio of 1.1 and cycled at 0.2 C (i.e., formation cycle) prior to the fast-charging test. Figure 4a presents the state of charge (SOC) vs. time profiles of full cells measured upon charging at 6 C. The cell was charged using a constant current (CC)–constant voltage (CV) protocol, i.e., CC charging at 6 C to 4.2 V, followed by CV charging with a 0.1 C cut-off current. The full cell with Mo-CP/graphite could be fully charged in 18.2 min, i.e., in a remarkably shorter time than the full cell with pristine graphite (23.6 min). Moreover, for Mo-CP/graphite, the charging time required to reach 80% SOC was estimated as 7.7 min, which was much lower than the USABC target (15 min) set for commercial LIBs^5^. To further demonstrate the fast-charging capability of the cycled full cells, the relative contributions of CC and CV charging modes to the total charging time were measured after 100 cycles, as shown in the inset of Fig. 4a. Note that the full cell with Mo-CP/graphite maintained a much larger contribution of the CC charging mode (34.3%) compared with that of pristine graphite (20.5%). In addition, the total charging times and capacities of full cells with pristine graphite and Mo-CP/graphite at selected cycles (1st, 50th, and 100th) were compared in Supplementary Table 5. More importantly, unlike for the pristine graphite anode, no Li plating occurred on the Mo-CP/graphite anode under the conditions of fast charging, as discussed below.

To examine the Li plating behaviours of different anodes in detail, we constructed three-electrode full cells: a Li reference electrode was incorporated between the anode and the cathode to perform independent measurements of electrode voltages. Figure 4b, c presents the voltage vs. SOC profiles of anodes and cathodes in full cells containing pristine graphite and Mo-CP/graphite, respectively, during charging at 0.5 C and 6 C. The anode voltage gradually decreased during CC charging and then slowly increased because of current reduction during CV charging. Both of the full cells showed the anode voltages higher than 0 V vs. Li/Li^+^ during charging at 0.5 C. The voltage of the pristine graphite anode rapidly dropped to below 0 V vs. Li/Li^+^ at 6 C, which possibly caused Li plating and incomplete lithiation. On the other hand, the voltage of the Mo-CP/graphite anode remained higher than 0 V vs. Li/Li^+^ throughout the charging period. This behaviour indicated that no Li plating would occur on the Mo-CP/graphite anode even at 6 C, which was confirmed by the full-cell cycling test (6 C charging/1 C discharging).

Figure 4d displays Nyquist plots obtained for pristine graphite and Mo-CP/graphite anodes at 0.3 V vs. Li/Li^+^, revealing a considerable reduction of interfacial resistance in the latter case. The electrode potential was so chosen that the impedance measurements could capture properly the contributions of Mo-CP to the interfacial kinetics of the anode without any complications arising from fully lithiated graphite. To clearly differentiate the elemental reaction steps involved in the lithiation process, impedance spectra were analysed by a distribution of relaxation times (DRT) technique^33,34^. Figure 4e presents plots of the DRT function (*γ*(*τ*)) vs. relaxation time (*τ*) obtained based on the impedance data, revealing that whereas reaction processes could be clearly distinguished in the DRT curve, they overlapped in the Nyquist-type impedance plot. As indicated in Fig. 4e, three characteristic peaks of reaction processes (*R*_1_, *R*_2_, and *R*_3_) were identified for pristine graphite in the *τ* range of 10^–1^ to 10^–6^ s. Peak *R*_3_ was attributed to the charge-transfer reaction at graphite, while peaks *R*_1_ and *R*_2_ at shorter relaxation times were ascribed to interfacial reactions, i.e., Li^+^ desolvation and subsequent migration through the solid-electrolyte interphase (SEI)^35,36^. The peak at *τ* > 10^–1^ s corresponds to Li diffusion in bulk graphite. Notably, compared to those of pristine graphite, the *γ*(*τ*) peaks of Mo-CP/graphite appeared at shorter *τ* values, which suggested that interfacial reactions were promoted by Mo-CP. Based on the temperature dependence of impedance, the activation energies (*E*_a_) for interfacial desolvation/migration reactions (*R*_1_ + *R*_2_) were estimated as ~60 and ~29 kJ mol^–1^ for pristine graphite and Mo-CP/graphite, respectively (Fig. 4f). The lower *E*_a_ value of the latter agreed with DFT calculation-determined ability of MoP_*x*_ to effectively lower the Li^+^ adsorption energy and thus facilitate Li^+^ insertion into graphite.

**Fig. 5:**
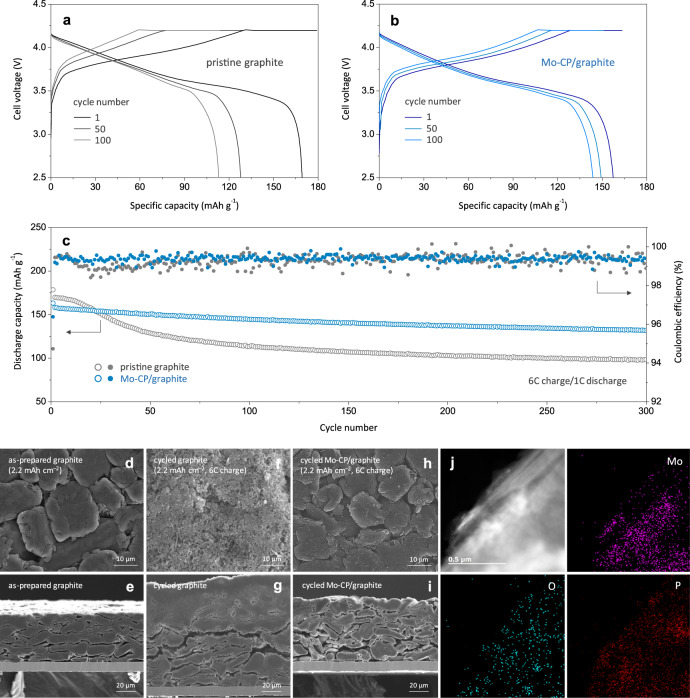
Cycling performance of Mo-CP/graphite under fast-charging conditions. **a**, **b** Voltage profiles of full cells assembled with pristine graphite (**a**) and 2 wt% Mo-CP/graphite (**b**) anodes at selected cycles. **c** Capacity decay and CEs of full cells with pristine graphite and Mo-CP/graphite anodes. Cycling was performed at charge/discharge rates of 6 C/1 C. **d**–**i** Surface (**d**, **f**, **h**) and cross-sectional (**e**, **g**, **i**) FESEM micrographs of as-prepared graphite (**d**, **e**), cycled graphite (**f**, **g**), and cycled Mo-CP/graphite (**h**, **i**) anodes. **j** TEM and EDS mapping results of Mo-CP/graphite after 300 cycles.

Figure 5a, b present galvanostatic charge–discharge profiles of full cells with pristine graphite and Mo-CP/graphite anodes, respectively, at selected cycles. At this point, it is worth noting that the voltage profiles of full cells with pristine graphite and Mo-CP/graphite were almost identical. That is, the introduction of a small amount of Mo-CP did not significantly reduce energy density, and the slight difference of voltage profiles was ascribed to the higher Li^+^ intercalation/deintercalation potentials observed in the presence of MoP_*x*_. During cycling, the capacity of the full cell with the pristine graphite anode rapidly decreased to ~66% after 100 cycles (Fig. 5a). Compared to the pristine graphite anode, the Mo-CP/graphite anode exhibited more stable cycling behaviours under fast-charging conditions, featuring capacity retentions of ~91% after 100 cycles (Fig. 5b). The stable cyclability of Mo-CP/graphite was further confirmed at various discharge rates (2 C and 3 C) (Supplementary Fig. 10). Moreover, the rate performance study (Supplementary Fig. 11) demonstrated the superior rate capability and capacity retention of the full cell with Mo-CP/graphite at 0.2 C–10 C, as compared with the full cell with pristine graphite. The Coulombic efficiency (CE) is an important performance descriptor for LIBs that serves as a measure of charge–discharge reversibility and as an indicator for Li plating^37,38^. Figure 5c shows the capacity retentions and CEs of full cells with pristine graphite and Mo-CP/graphite with an areal capacity of 2.2 mAh cm^–2^ (6 C charging/1 C discharging). During the first 20 cycles, the CE of the full cell with pristine graphite was very low, because of the occurrence of metallic Li plating and continuous SEI growth, which led to a significant loss of charge capacity. On the other hand, even during fast-charging cycles, the full cell with the Mo-CP/graphite anode showed high CE values of ~99.8%, complying with the commercial requirement. Moreover, it showed cycling stability with a capacity retention of ~84% even after 300 cycles (Fig. 5c). The cycling performance of full cells with Mo-CP/graphite with areal capacities of 3.2 and 4.0 mAh cm^–2^ (3 C charging/1 C discharging) was also examined and presented in Supplementary Fig. 12. The full cells with the Mo-CP/graphite anodes exhibited higher capacity retentions, i.e., ~91% (3.2 mAh cm^–2^) and ~64% (4.0 mAh cm^–2^), after 50 cycles compared to those of pristine graphite. At this point, it should be noted that high-capacity electrodes suffer from large ion-transport limitation in the internal pores (Supplementary Note 5), as indicated by a higher MacMullin number (17.3) for 3.2 mAh cm^–2^ than that (12.1) for 2.2 mAh cm^–2^ (Supplementary Fig. 13). Therefore, comprehensive engineering work is further required to design and optimise the microstructures of high-capacity electrodes to make them fast-chargeable. Supplementary Table 6 compares the fast-charging performance of the Mo-CP/graphite anode developed in this work with those of various anode materials reported in the literature and clearly demonstrates a significant improvement in the fast-charge cyclability of LIBs achieved in this study.

After 300 fast-charging cycles, full cells were disassembled, and the morphological and structural changes of their anodes were examined. Figure 5d–i shows top-view and cross-sectional FESEM images of as-prepared (fresh) and cycled anodes. Each anode was exposed to ambient atmosphere prior to EDS analysis (Supplementary Figs. 14 and 15), and the oxygen signal was recorded to determine the spatial distribution of LiO_*x*_^39^. In contrast to the case of the as-prepared graphite anode (Fig. 5d, e), severe growth of metallic Li dendrites (thickness ~50 μm) was observed on the surface of the cycled graphite anode (Fig. 5f, g, Supplementary Figs. 14 and 16a), which confirmed that fast-charging cycling of the graphite anode was accompanied by continuous undesirable Li plating. It should be noted that the cycled Mo-CP/graphite anode had clean surfaces free of metallic Li dendrites (Fig. 5h, i, Supplementary Fig. 16b) in good agreement with the charging behaviour of this electrode (Fig. 4c). The critical role of Mo-CP in suppressing undesirable Li plating on graphite was further confirmed using a multi-layer pouch-type full cell (Supplementary Fig. 17). The dimensional stability of the Mo-CP nanolayer is of potential concern, because MoP_*x*_ may undergo conversion-type lithiation reactions. Given the fact that the reaction mechanism for lithiation strongly depends on the crystallite size, the nanocrystalline MoP_*x*_ phase with a large fraction of imperfect bonds at surfaces would undergo insertion-type lithiation reactions that are different than conversion-type lithiation observed for bulk-materials, as has been previously reported^27,40,41^. Although MoP_*x*_ powders synthesised by a solid-state method suffered from a volume change of ~24% upon lithiation (Supplementary Fig. 7), the volume variations of nanoscale MoP_*x*_ dispersed in the MoO_*x*_ matrix would be small and could be effectively accommodated, if any. In fact, the post-mortem TEM analysis confirmed that the morphologies of Mo-CP/graphite remained almost unchanged even after 300 cycles (Fig. 5j, Supplementary Fig. 18).

Taking the requirements for practical LIBs into account, we proposed the design of fast-chargeable anodes based on a cooperative biphasic MoO_*x*_–MoP_*x*_ promoter. A comprehensive electrochemical study combined with DFT calculations and in situ measurements demonstrated that MoP_*x*_ dispersed in the MoO_*x*_ layer is involved in a fast Li^+^ intercalation reaction at relatively high potentials (~0.7 V vs. Li/Li^+^) while simultaneously reducing overpotentials arising from the desolvation and adsorption of Li^+^ at the surface. Moreover, the conformal MoO_*x*_ nanolayer effectively mitigates the formation of resistive films on the graphite surface. The efficacy of Mo-CP for improving the fast-charging performance and suppressing Li plating was fully demonstrated in a full cell. Furthermore, the dimensional stability of Mo-CP was shown to secure the stable performance of anode materials over 300 cycles. Thus, our study suggests that surface engineering of graphite with Mo-CP is a promising way of improving the fast-charging capability and cycle lifetime of LIBs. Given that the synthesis of Mo-CP/graphite via vapour-induced phase transformation is easy to scale up for commercial production, the proposed technology may bring forward the successful realisation of high-energy-density and fast-chargeable LIBs.

## Methods

### Material synthesis

To prepare Mo-CP/graphite, MoO_3_ powder (0.2 g, 99.9%, Sigma-Aldrich) was dissolved in H_2_O_2_ (40 mL, 35 wt%, Daejung), and the obtained solution was mixed with graphite powder (9.8 g) (Supplementary Table 7) under continuous stirring. The solvent was removed overnight at 80 °C to afford MoO_*x*_-deposited graphite powder, which was then placed at the centre of a tube furnace and heated at 600 °C upon the continuous feeding of P-containing gas generated by the thermal decomposition of NaH_2_PO_2_ powder (10 g, 99.0%, Sigma-Aldrich) (Supplementary Fig. 19). After 2 h, the resulting Mo-CP/graphite was cooled down to room temperature and carefully ground before use.

### Structural characterisation

Morphologies and microstructures were characterised by FESEM (JEOL, JSM-7000F) and TEM (JEOL, ARM-200F, 200 kV) coupled with EDS. Powder XRD patterns were obtained using an X-ray diffractometer (Empyrean, PANalytical) with Cu *K*_α_ radiation (*λ* = 0.154046 nm). XPS (Thermo Scientific, K-alpha) was employed to investigate the chemical states of materials. In situ XRD and ex situ XPS analyses were carried out for MoP_*x*_ with different SOCs to investigate structure evolution during Li intercalation and deintercalation. Mercury intrusion porosimetry (Micromeritics, AutoPore IV 9510) was employed to measure the porosity of the electrodes.

### Electrochemical measurements

For electrochemical performance evaluation, electrodes were fabricated by a conventional slurry coating process. A slurry prepared by mixing active material (96 wt%) with polyvinylidene fluoride (PVdF) binder (4 wt%) in *N*-methylpyrrolidone was coated on Cu foil (current collector), dried at 120 °C for 12 h, and the electrodes were pressed to achieve an electrode density of 1.5 g cm^–3^. The amount of anode material was carefully controlled to reach areal capacities of 2.2, 3.2, and 4.0 mAh cm^–2^. For coin-type, three-electrode half-cell (CR2032) assembly, the anode material was used as the working electrode, while Li was employed as counter and reference electrodes. For coin-type full-cell (CR2032) assembly, a commercial NCM cathode was prepared on Al foil (current collector) through a conventional slurry coating technique using a fixed composition of 94 wt% NCM: 3 wt% conducting agent (Super-P): 3 wt% binder (PVdF). The full cell was designed with an *N*/*P* ratio of 1.1. The areas of the anode and cathode were 1.54 and 1.13 cm^2^, respectively. A polyethylene membrane was used as a separator and 1 M LiPF_6_ dissolved in a mixture of ethylene carbonate: ethylmethyl carbonate (3:7, v/v) was used as an electrolyte without any additives. The electrolyte amount was 100 μL. Cells were carefully assembled in an Ar-filled glove box. For three-electrode measurements, a Li reference electrode was placed between two separators, and cells were assembled using the materials and procedure used for full cells. Full cells were galvanostatically charged and discharged in a voltage range of 2.5–4.2 V at a constant current density of 0.2 C for three cycles and then further charged at either 6 C or 3 C and discharged at 1 C. Electrochemical impedance spectra were recorded at 0.3 V vs. Li/Li^+^ in a temperature range of 5–35 °C using a Bio-Logic SP-240 impedance analyser. To investigate Li plating behaviour, full cells were carefully disassembled in an Ar-filled glove box, and the cycled electrodes were collected and characterised.

### DFT calculations

All DFT calculations were performed using projector-augmented wave pseudopotentials implemented in the Vienna ab initio simulation package^42,43^. The generalised Perdew–Burke–Ernzerhof gradient approximation was employed for exchange-correlation functional parameterisation^44^. Li pseudopotential was calculated using one 2 s and two 1 s electrons as valence electrons to more precisely describe Li^+^ ions. For standard computational parameters, a 5 × 5 × 5 and 5 × 1 × 5 *k*-point meshes in the Monkhorst-Pack scheme were set for bulk and surface calculations, respectively. The energy cut-off for the plane-wave basis point was set to 500 eV for the structural optimisation of both the bulk unit cell and the surface model. All internal coordinates were relaxed until the force acting on each atom was less than 0.01 eV Å^–1^. The formation energies of interstitial Li into bulk MoP and MoP_2_ and Li^+^ adsorption energies on the surfaces of MoP and MoP_2_ were calculated as follows:

1 $$\Delta E_{\mathrm{f}} = E_{{\mathrm{Li}}_x{\mathrm{MoP}}_2} - \left[ {E_{{\mathrm{MoP}}_2} + x \cdot E_{{\mathrm{Li}}}} \right]\;\left( {{\mathrm{for}}\;{\mathrm{MoP}}_2} \right),$$

where *E*_MoP2_ and *E*_Li_ are the total free energies of the bulk (or surface) MoP_2_ and Li metal (bulk), respectively.

## Supplementary information

Supplementary Information

## Data Availability

The data that support the findings of this study are available from the corresponding author upon reasonable request.
