# Supplementary material for: A cooperative biphasic MoOx–MoPx promoter enables a fast-charging lithium-ion battery
Source: Nat Commun. 2021 Jan 4;12:39. doi: 10.1038/s41467-020-20297-8 (PMC7782533; doi:10.1038/s41467-020-20297-8)
Supplement: Supplementary file 1 — Supplementary Information [file 41467_2020_20297_MOESM1_ESM.pdf]

Supplementary Information

A cooperative biphasic MoO_x–MoP_x promoter enables a fast-charging lithium-ion battery

Sang-Min Lee [1], Junyoung Kim [2], Janghyuk Moon [3], Kyu-Nam Jung [4], Jong Hwa Kim [2], Gum-Jae Park [1], Jeong-Hee Choi [1], Dong Young Rhee [2], Jeom-Soo Kim [5], Jong-Won Lee [6] and Min-Sik Park [2]**

[1] Battery Research Center, Korea Electrotechnology Research Institute, 12 Bulmosan-ro 10 beon-gil, Changwon 51543, Republic of Korea

[2] Department of Advanced Materials Engineering for Information and Electronics, Kyung Hee University, 1732 Deogyeong-daero, Giheung-gu, Yongin 17104, Republic of Korea

[3] School of Energy System Engineering, Chung-Ang University, 84 Heukseok-ro, Dongjak-gu, Seoul 06974, Republic of Korea

[4] New and Renewable Energy Institute, Korea Institute of Energy Research, 152 Gajeong-ro, Yuseong-gu, Daejeon 34129, Republic of Korea

[5] Department of Chemical Engineering, Dong-A University, 37 Nakdong-daero, Saha-gu, Busan 49315, Republic of Korea

[6] Department of Energy Science and Engineering, Daegu Gyeongbuk Institute of Science and Technology (DGIST), 333 Techno Jungang-daero, Hyeonpung-eup, Dalseong-gun, Daegu 42988, Republic of Korea

* Corresponding authors

E-mails: jongwon@dgist.ac.kr (J.-W. Lee), mspark@khu.ac.kr (M.-S. Park)

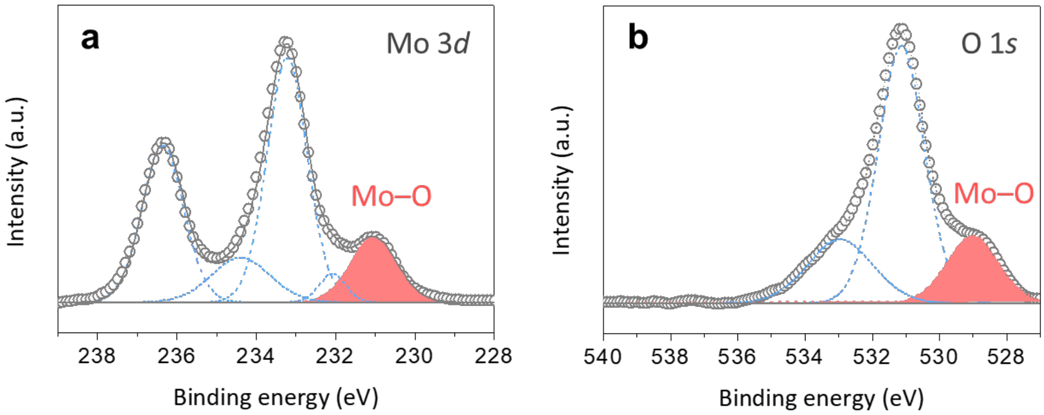

Supplementary Figure 1. Deconvoluted XPS spectra of graphite obtained after MoO_x coating.

Supplementary Note 1. DFT calculations for the lithiation of MoP and MoP_2

(a) Lithiation of MoP

A 2 × 2 × 2 supercell with eight MoP units was chosen to investigate Li insertion into MoP. Li atoms were inserted from 1 to 8 at different positions using the Delaunary triangulation method. The four expected Li insertion sites for $Li_{0.5}MoP$ formation were selected by considering the calculation results for all possible configurations (Supplementary Table 1). The parameters of fully relaxed $Li_{0.5}MoP$ structures are presented in Supplementary Table 2.

Supplementary Table 1. Atomic coordination in the unit cell of $Li_{0.5}MoP$.

Lattice constant (Å)	a<br>6.4812	b<br>6.4812	c<br>6.3862
Angle (°)	α<br>90	β<br>90	γ<br>120
Coordination	*x*	*y*	*z*
Mo1	0	0	0
Mo2	0.5	0	0
Mo3	0	0.5	0
Mo4	0.5	0.5	0
Mo5	0	0	0.5
Mo6	0.5	0	0.5
Mo7	0	0.5	0.5
Mo8	0.5	0.5	0.5
P1	0.1667	0.3333	0.25
P2	0.6667	0.3333	0.25
P3	0.1667	0.8333	0.25
P4	0.6667	0.8333	0.25
P5	0.1667	0.3333	0.75
P6	0.6667	0.3333	0.75
P7	0.1667	0.8333	0.75
P8	0.6667	0.8333	0.75
Li1	0.34	0.18	0.14
Li2	0.34	0.18	0.64
Li3	0.84	0.68	0.14
Li4	0.84	0.68	0.64

Supplementary Table 2. Calculated lattice parameters of MoP and $Li_{0.5}MoP$.

	Lattice constant (Å)			Angle (°)		
	a	b	c	α	β	γ
MoP (Ref)	3.231	3.231	3.207	90.00	90.00	120.00
Mo_4P_4	3.239	3.239	3.196	90.00	90.00	120.00
$Li_4Mo_8P_8$	7.064	7.064	6.307	90.01	90.00	121.80

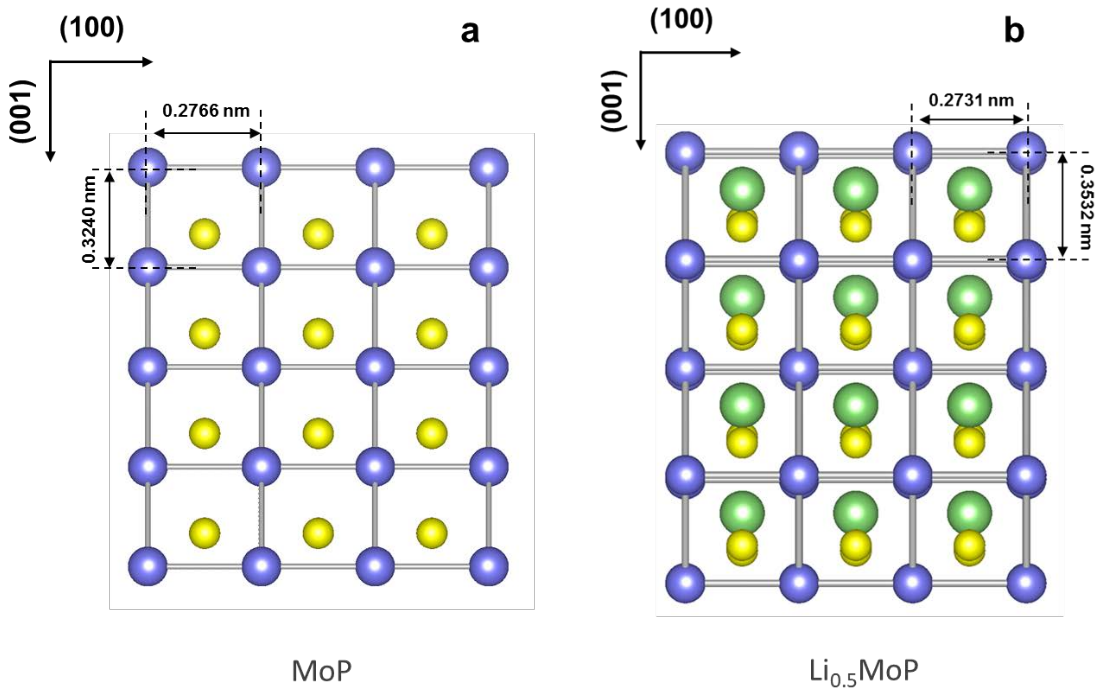

Supplementary Figure 2. Calculated crystal structures of (a) MoP and (b) $Li_{0.5}MoP$.

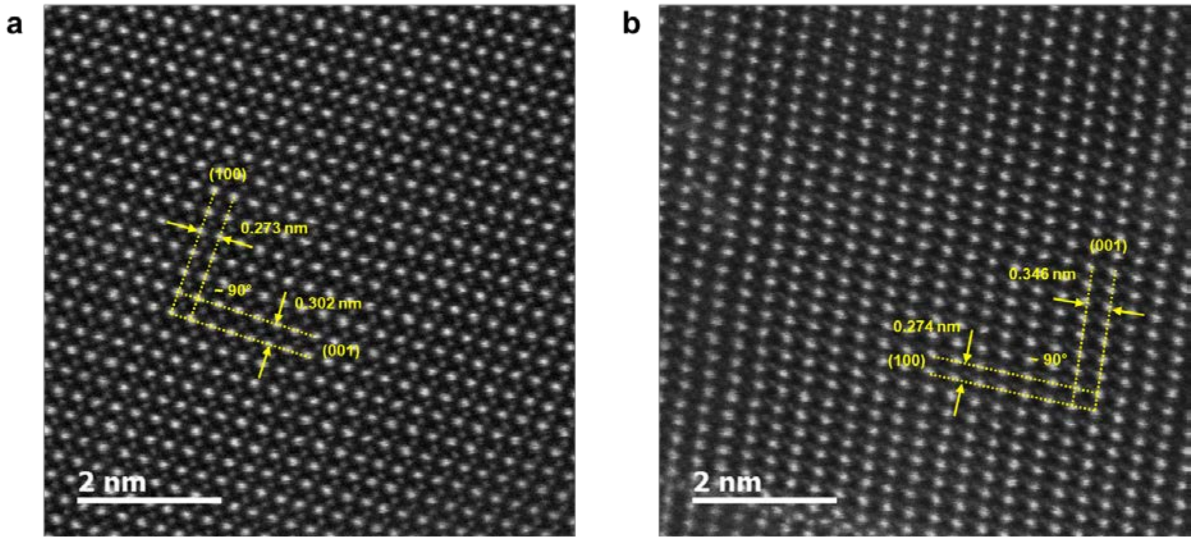

Supplementary Figure 3. TEM micrographs of (a) MoP and (b) $Li_{0.5}MoP$.

(b) Lithiation of MoP_2

In the case of MoP_2, the intermediate structure of $Li_{\delta}MoP_2$ ($\delta = 2$) was generated by Li intercalation into the basic structure of MoP_2. First, one Li atom was added onto eight potential locations with large spaces in the unit cell of MoP_2. Then, each structure was relaxed using DFT calculations to find the most stable position of Li in the structure. Finally, the resulting intermediate structures and corresponding formation energies were calculated during the sequential lithiation of MoP_2 to $Li_{\delta}MoP_2$ ($\delta = 2$), as shown in Supplementary Fig. 4 and Supplementary Table 3 and 4.

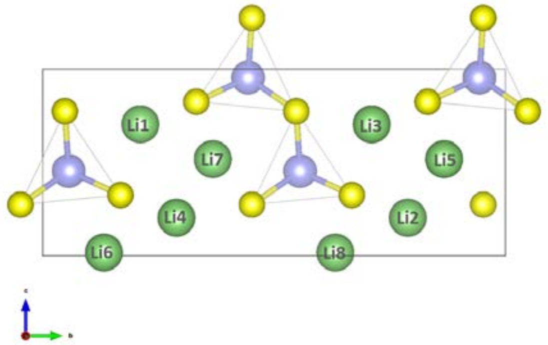

Supplementary Figure 4. Relaxed structure of Li_2MoP_2 obtained by Li^+ insertion and the corresponding sequence numbers.

Supplementary Table 3. Atomic coordination in the unit cell of Li_2MoP_2.

Lattice constant (Å)	a	b	c
	3.1332	15.3062	6.1709
Angle (°)	α	β	γ
	90	90	90
Coordination	*x*	*y*	*z*
Mo1	0	0.05618	0.45424
Mo2	0.5	0.44386	0.95416
Mo3	0.5	0.55623	0.45418
Mo4	0	0.94375	0.95416
P1	0	0.45205	0.27389
P2	0.5	0.04792	0.77399
P3	0.5	0.16808	0.32534
P4	0	0.33204	0.82508
P5	0.5	0.95202	0.27389
P6	0	0.54801	0.77391
P7	0	0.66806	0.32526
P8	0.5	0.83192	0.82511
Li1	0.5	0.21099	0.70424
Li2	0.5	0.78904	0.20387
Li3	0	0.71098	0.70397
Li4	0	0.28902	0.20373
Li5	0	0.86756	0.51766
Li6	0	0.13227	0.01795
Li7	0.5	0.36757	0.51761
Li8	0.5	0.63243	0.01777

Supplementary Table 4. Calculated lattice parameters of MoP_2 and Li_2MoP_2.

	Lattice constant (Å)			Angle (°)		
	a	b	c	α	β	γ
MoP_2 (Ref)	3.15861	11.24906	5.01066	90.00	90.00	90.00
MoP_2	3.16431	11.23673	4.99163	90.00	90.00	90.00
$Li_8Mo_4P_8$	3.13318	15.30619	6.17087	90.01	90.00	90.00

Supplementary Note 2. Synthesis and characterisation of MoP_x particles

MoP_x particles were synthesised by a solid-state method. Mo and red-P precursors with different weight ratios were mechanically milled at 1000 rpm for 12 h in a stainless steel jar to obtain MoP_x crystallites interconnected on the nanoscale. The resulting powders were collected and sieved before use. With an increase in the amount of red-P, the fraction of MoP_2 phase in MoP_x gradually increased, as shown in the XRD patterns of MoP_x with x = 1.2 and 1.6 (Supplementary Fig. 5a). We found an optimum composition to be x = 1.6 in terms of the reversible capacity, the Li^+ adsorption energy, and the volume expansion upon lithiation in this work. Powder XRD analysis confirmed that MoP_x particles comprised hexagonal MoP (JCPDS 24-0771, *P6m2* space group) and orthorhombic MoP_2 (JCPDS 16-0499, *Cmc21* space group)[1,2] (Supplementary Fig. 5a). The morphology and microstructure of as-prepared MoP_x nanoparticles were investigated by FESEM and TEM, which revealed that irregular-shaped sub-micron-size MoP_x particles comprised aggregates of interconnected nano-crystallites (~10 nm) (Supplementary Fig. 5b and c). The chemical composition of MoP_x nanoparticles was further confirmed by EDS (Supplementary Fig. 5d and e).

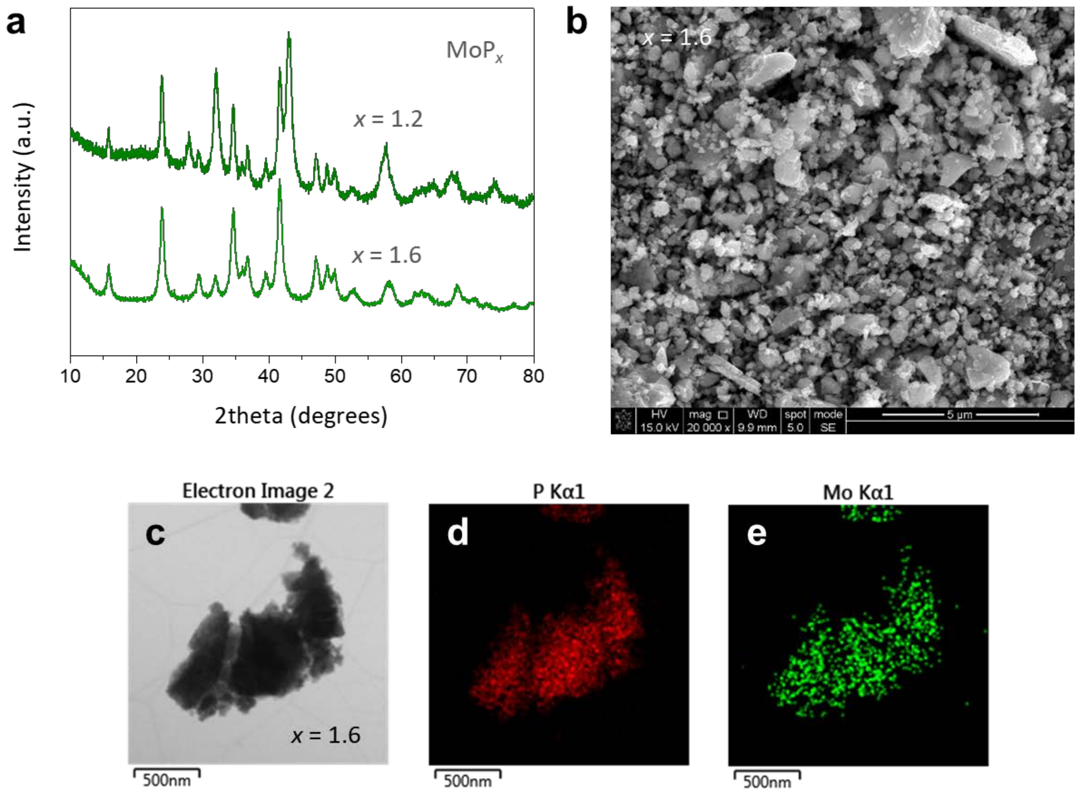

Supplementary Figure 5. (a) XRD patterns, (b) SEM image, and (c) TEM image and (d,e) corresponding EDS mappings of MoP_x.

Supplementary Note 3. Electrochemical behaviour of MoP_x

The lithiation–delithiation behaviour of MoP_x was examined using a galvanostatic intermittent titration technique (GITT) with a current density of 20 mA g^{-1}, as shown in Supplementary Fig. 6a. Here, MoP_x electrodes were fabricated by coating a slurry of active material (80 wt%), Super-P (10 wt%), and polyvinylidene fluoride (PVdF) (10 wt%) in *N*-methylpyrrolidone on Cu foil. The GITT profiles confirmed that MoP_x was lithiated at higher voltages than graphite. The initial Coulombic efficiency was estimated to be as low as ~77% (Supplementary Fig. 6b) at 20 mA g^{-1}; however, considering the low loading of Mo-CP (2 wt%) in the Mo-CP/graphite, the Li loss during the first cycle would be negligibly small. After the first cycle, MoP_x exhibited a charge capacity of 533 mAh g^{-1} at 200 mA g^{-1} and showed no capacity decay over 90 cycles.

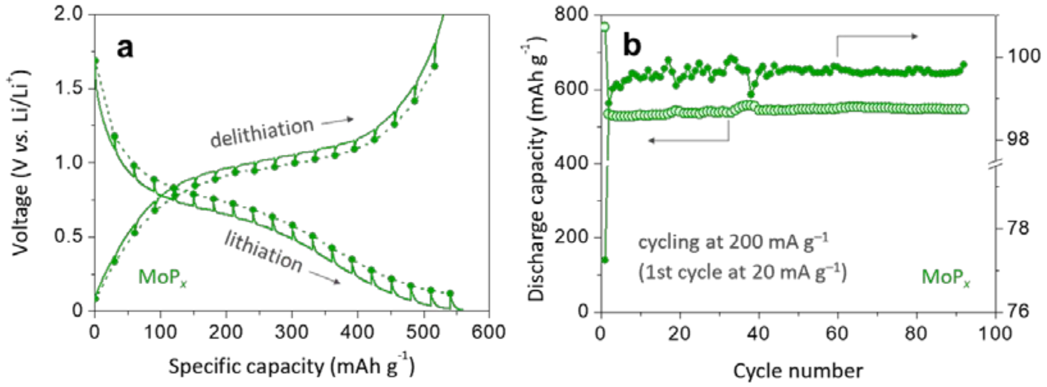

Supplementary Figure 6. Electrochemical behaviour of MoP_x particles: (a) GITT profile and (b) cycling performance at 200 mA g^{-1}.

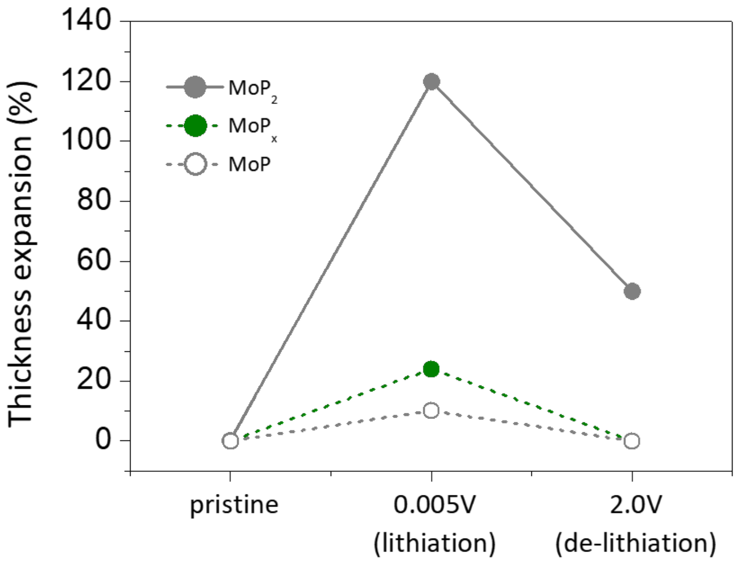

Supplementary Figure 7. Thickness variation of MoP_x electrodes upon lithiation/delithiation.

Supplementary Note 4. Characterisation of SEIs

To avoid undesirable Li plating and dendritic growth of metallic Li at the surface of graphite under fast-charging conditions, it is necessary to form an SEI layer at the surface of graphite. Since the formation of the SEI layer is spontaneously induced by the decomposition of electrolytes at the surface of graphite, the chemical composition of the SEI layer is mainly affected by the electrolyte and the surface chemistry of graphite. Supplementary Fig. 8 compares the XPS spectra collected from pristine graphite, MoO_x/graphite and Mo-CP/graphite anodes after the formation cycles. The XPS spectra were carefully de-convoluted based on the C 1*s* excitation at a binding energy of 284.5 eV. In the Li 1*s* spectra, characteristic peaks for Li_2CO_3 and LiF were dominantly observed at 55.5 and 56.4 eV, respectively, together with traces of Li_2O (53.9 eV) and Li_3P (57.7 eV). The results indicate that the formation of resistive Li_2CO_3 species was effectively reduced on MoO_x/graphite and Mo-CP/graphite because the direct exposure of carbon surface could be minimised by the surface modifications with the conformal MoO_x coating.

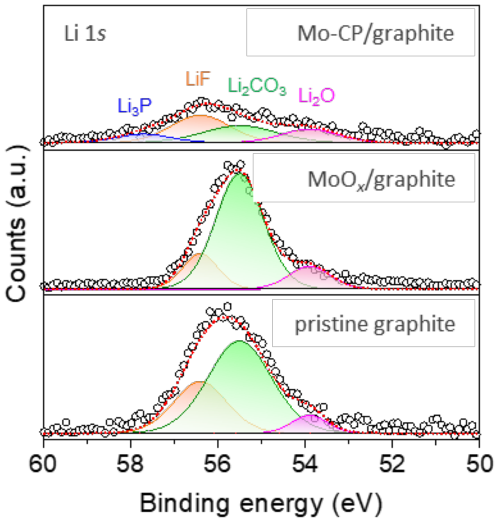

Supplementary Figure 8. XPS Li 1*s* spectra of pristine graphite, MoO_x/graphite and Mo-CP/graphite anodes measured after the formation cycles.

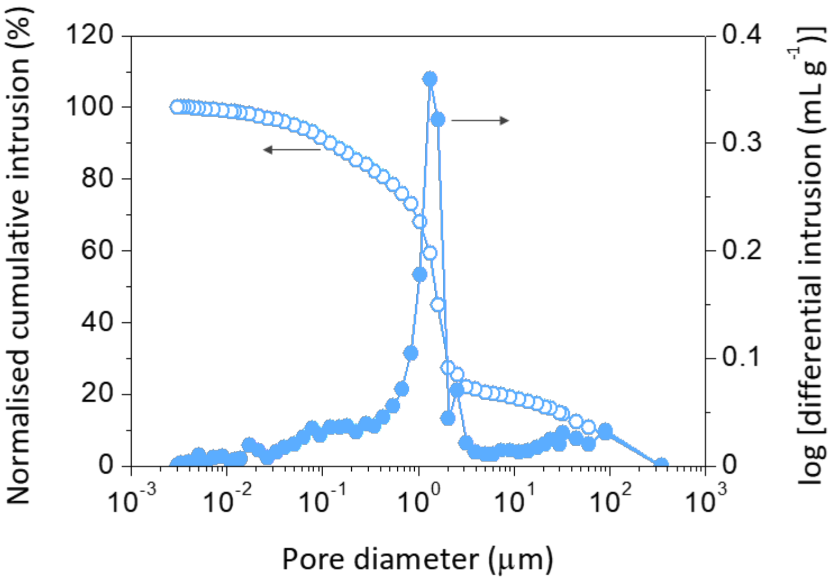

Supplementary Figure 9. Normalised cumulative intrusion and pore size distribution for the Mo-CP/graphite electrode (2.2 mAh cm^{-2}) measured by mercury intrusion porosimetry. The porosity was estimated to be 35.0%.

Supplementary Table 5. Total charging times and charge capacities of full cells with pristine graphite and Mo-CP/graphite anodes.

Anode in full cell	Cycle number	Time for 100% SOC (min)	Charge capacity (mAh g^{-1})
Pristine graphite	1	23.6	179.1
	50	18.7	129.1
	100	17.5	113.7
Mo-CP/graphite	1	18.2	163.0
	50	17.6	151.0
	100	18.5	145.3

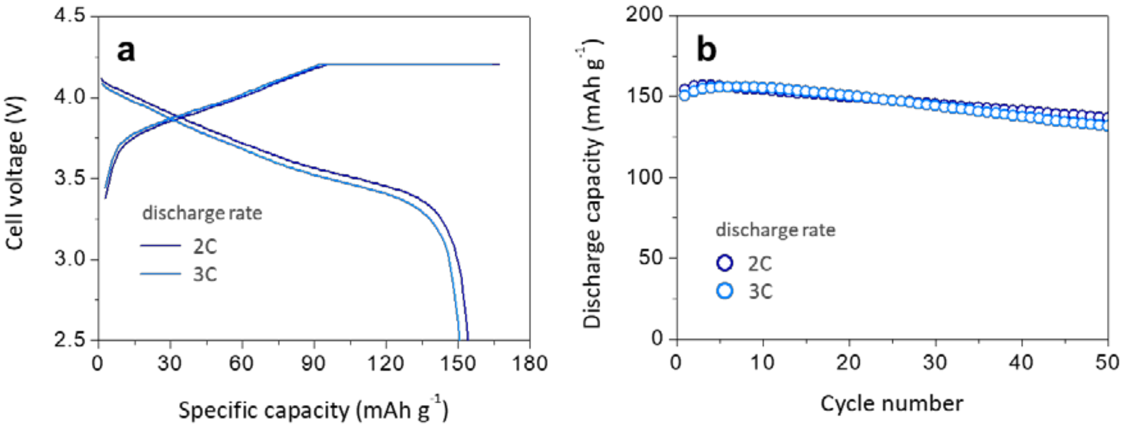

Supplementary Figure 10. (a) First cycle voltage profiles and (b) cycling performance of the full cell assembled with Mo-CP/graphite (2.2 mAh cm^{-2}). Cycling was performed at a charge rate of 6C and different discharge rates (2C and 3C).

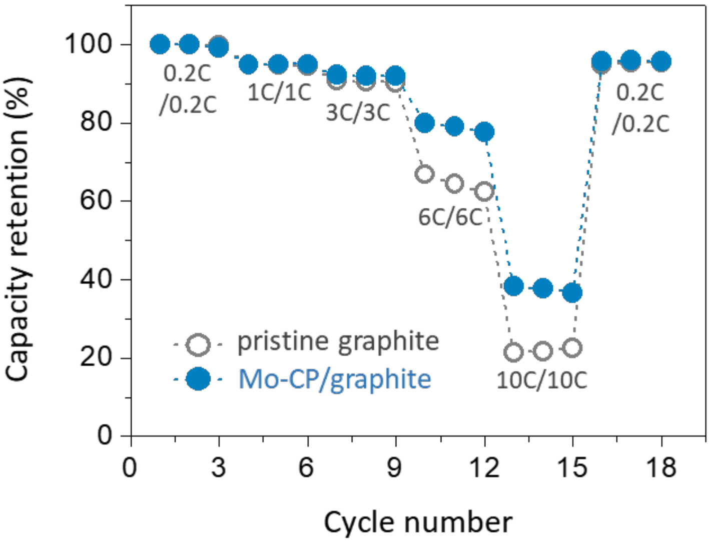

Supplementary Figure 11. Rate performance of full cells assembled with pristine graphite and Mo-CP/graphite (2.2 mAh cm^{-2}). Cycling was performed at various rates of 0.2C–10C during both charging and discharging.

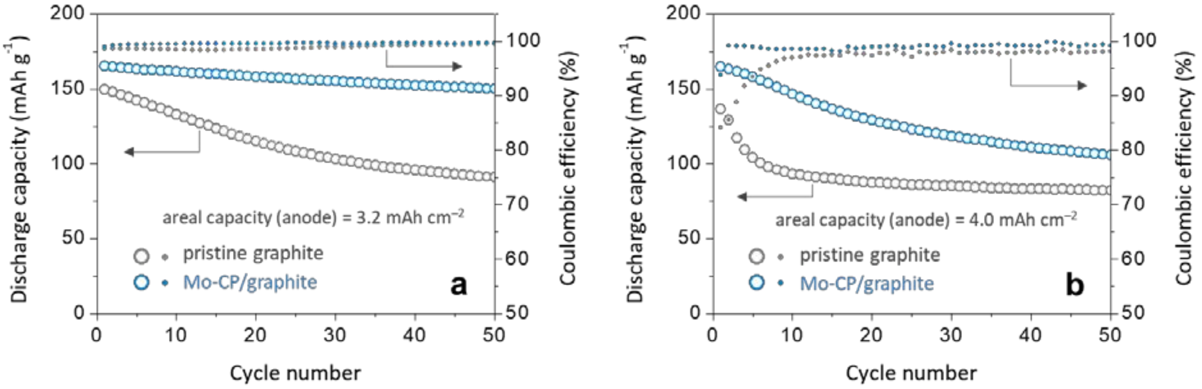

Supplementary Figure 12. Capacity decay and CEs of full cells constructed using pristine graphite and Mo-CP/graphite anodes with areal capacities of (a) 3.2 mAh cm^{-2} and (b) 4.0 mAh cm^{-2}. Cycling was performed at charge/discharge rates of 3C/1C.

Supplementary Note 5. Estimation of MacMullin numbers of the electrodes

The MacMullin number (N_M) is defined as the ratio of the ionic conductivity (κ) of an electrolyte solution to the effective ionic conductivity (κ_{eff}) of an electrolyte-impregnated electrode, and thus, it represents a measure of the ionic transport limitation in the porous electrode[3]. Supplementary Figure 11a and b present Nyquist plots obtained for Mo-CP/graphite anodes with the areal capacities of 2.2 and 3.2 mAh cm^{-2}, respectively, in a mixture of ethylene carbonate : dimethyl carbonate (5:5, v/v) solution containing 50 mM tetrabutylammonium-PF_6 ($TBAPF_6$). A symmetric cell was used to measure the impedance. The AC-impedance spectra consisted of a straight line inclined at a constant phase angle to the real axis at high frequencies (semi-infinite ion migration in the internal pores) and an almost vertical capacitive line at low frequencies (ion accumulation at the bottom of the pores). The measured impedance (Z) spectra were fitted using the generalised transmission-line model, and the values of N_M were estimated to be 12.1 for 2.2 mAh cm^{-2} and 17.3 for 3.2 mAh cm^{-2} from the ionic resistance (R_{ion}) and the area (A) and thickness (d) of the electrode:

$$Z(\omega) = R_{\text{ion}} \frac{\coth[(j\omega T)^{\alpha}]}{(j\omega T)^{\alpha}} \quad \text{(Supplementary Equation 1)}$$

$$N_M = \frac{R_{\text{ion}} A \kappa}{2d} \quad \text{(Supplementary Equation 2)}$$

where j is the complex number, ω is the angular frequency, T and α mean the fitting parameters characterizing ion transport, and A and d represent the dimensional area and thickness of the electrode, respectively.

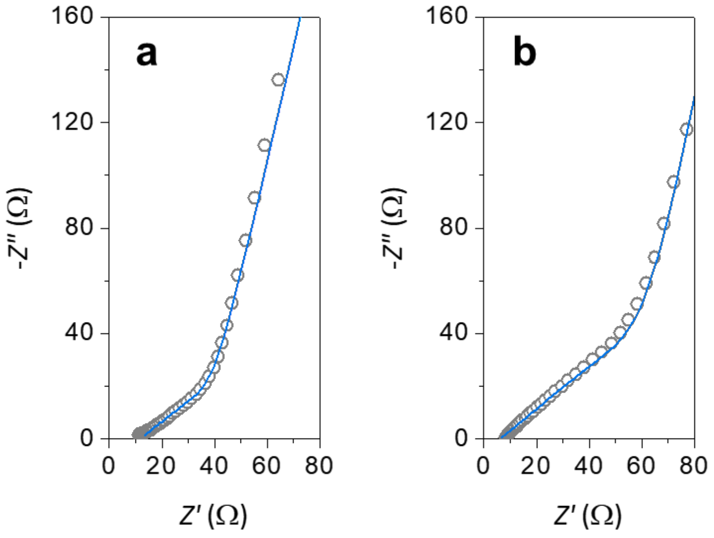

Supplementary Figure 13. AC-impedance spectra of the Mo-CP/graphite electrodes with the areal capacities of (a) 2.2 mAh cm^{-2} and (b) 3.2 mAh cm^{-2} measured in 50 mM $TBAPF_6$ in EC/DMC. Solid lines represent the results of fitting based on Supplementary Equation 1.

Supplementary Table 6. Comparison of the fast chargeability of various anode materials for LIBs.

Anode material	Half cell (with Li counter electrode)									Full cell					Ref.
	Electrode composition (wt%)			Mass loading (mg cm^{-2})	Areal capacity (anode) (mAh cm^{-2})	ICE[d] (%)	Charging current density (mA cm^{-2})	Fast chargeability[e] (%)	Cathode material	N/P ratio (-)	Areal capacity (cathode) (mAh cm^{-2})	Charging current density (mA cm^{-2})	Fast-charge cyclability (retention) (%@cycle no.)		
	AM[a]	CA[b]	BM[c]												
Si/edge-activated graphite	96	1	3	10.0	3.50	93.8	7.00	20.5	LCO[f]	1.00	3.40	10.2	67@50		4
KOH-etched graphite	96	1	3	5.0	1.74	92.4	8.71	13.8	-	-	-	-	74@100		5
VGCF/carbon nano-horn/graphite	95	0	5	6.0	2.03	84.0	6.09	7.00	-	-	-	-	-		6
Magnetically aligned graphite flake	80	10	10	5.0	1.49	-	2.98	17.4	-	-	-	-	-		7
Graphene-like-graphite	92	3	4	5.0	2.80	56.0	28.0	32.1	NCM111[g]	2.00	1.40	8.40	79@100		8
C@SnO_2@C	80	10	10	1.0	0.81	64.6	8.00	-	-	-	-	-	-		9
Sn/C hollow sphere	70	10	20	0.8	0.56	80.0	8.00	-	-	-	-	-	-		10
$Nb_{16}W_5O_{55}$ / $Nb_{18}W_{16}O_{93}$	80	10	10	2.0	0.27	-	5.49	-	-	-	-	-	-		11
3DOM-$TiNb_2O_7$	70	20	10	1.5	0.41	84.0	4.06	-	-	-	-	-	-		12
Li_3VO_4/C	82	8	10	-	-	79.3	-	-	-	-	-	-	-		13
Carbon nanosheet	80	10	10	0.8	0.56	50.0	4.00	-	-	-	-	-	-		14
Multi-channel graphite	97	1	2	6.5	2.30	94.0	13.8	-	-	-	-	-	-		15
Mo-CP/graphite	96	0	4	6.8	2.20	92.0	13.2	28.7	NCM622[h]	1.10	2.00	12.0	84@300		This work
				10.8	3.52				NCM622[h]	1.10	3.20	9.60	86@100		

[a] Active material; [b] Conducting agent; [c] Binder material; [d] Initial coulobmic efficiency; [e] Capacity delivered under fast-charging condition with respect to the nominal capacity; [f] $LiCoO_2$;

[g] $LiNi_{1/3}Co_{1/3}Mn_{1/3}O_2$; [h] $LiNi_{0.6}Co_{0.2}Mn_{0.2}O_2$

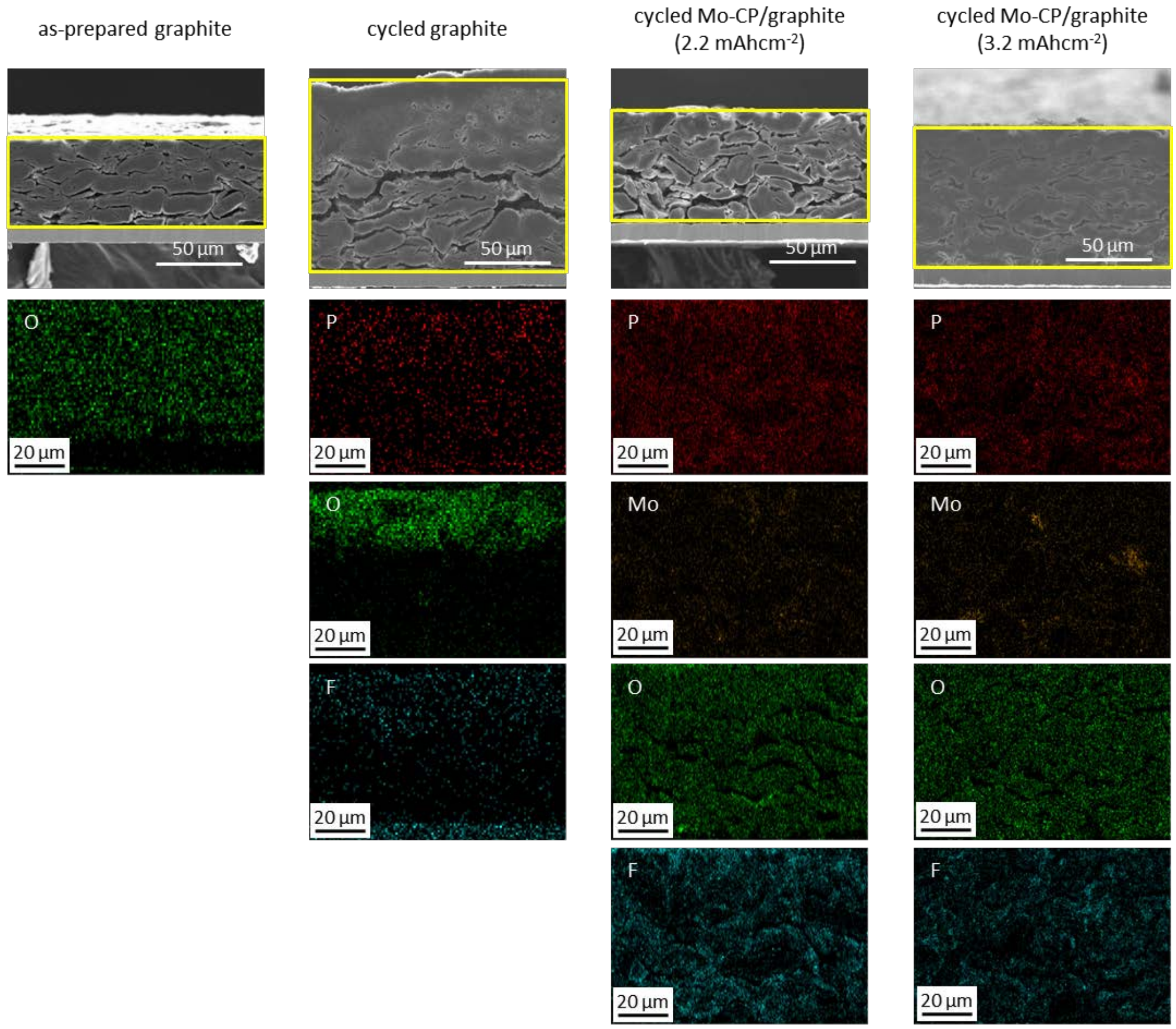

Supplementary Figure 14. EDS mappings of pristine graphite and Mo-CP/graphite anodes subjected to 100 cycles.

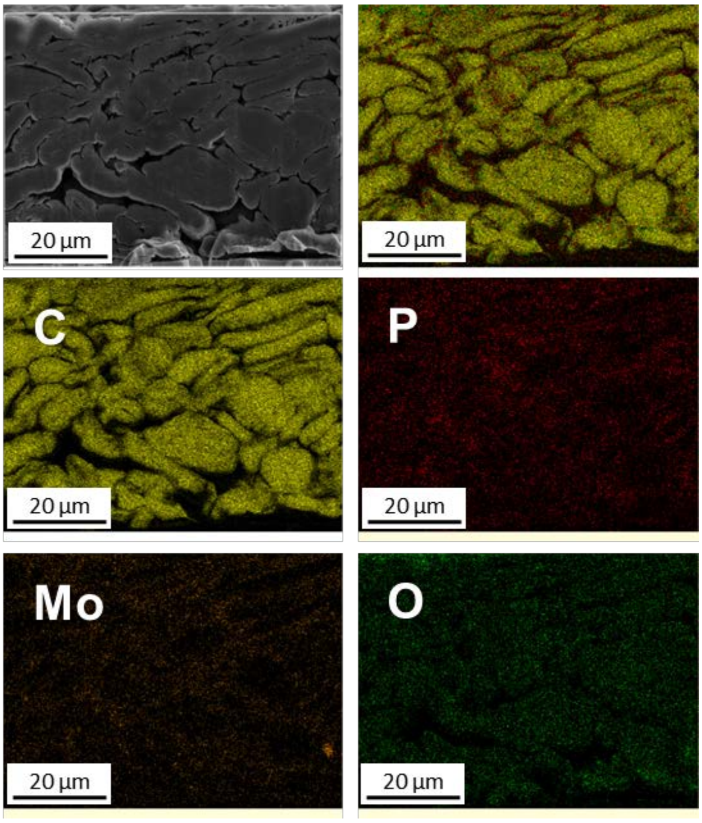

Supplementary Figure 15. EDS mappings of the Mo-CP/graphite anode (2.2 mAh cm^{-2}) subjected to 300 cycles.

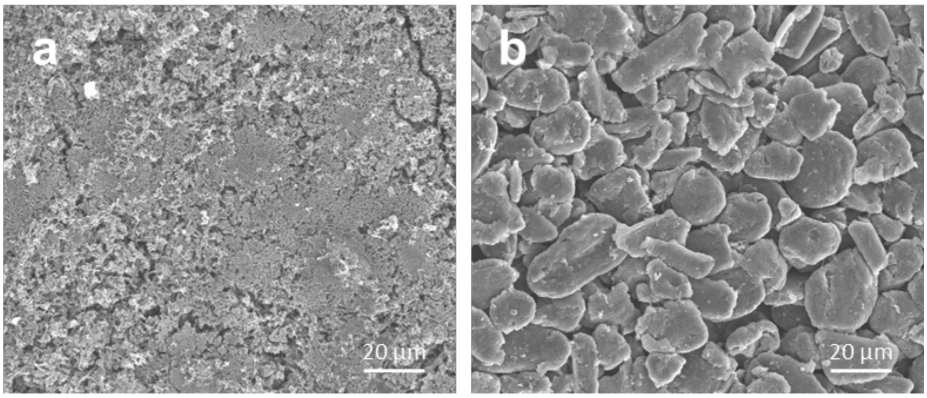

Supplementary Figure 16. SEM micrographs of (a) cycled graphite and (b) cycled Mo-CP/graphite anodes (4.0 mAh cm^{-2}). Cycling was performed at charge/discharge rates of 3C/1C.

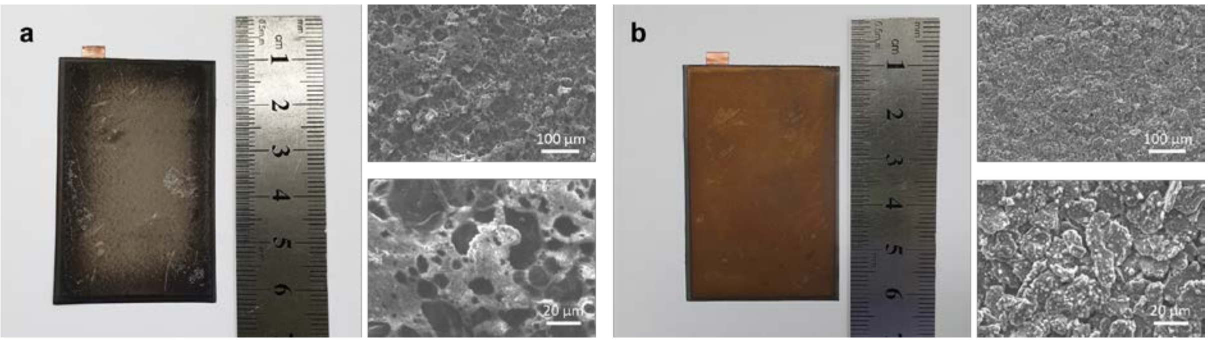

Supplementary Figure 17. Photographs and SEM images of (a) pristine and (b) Mo-CP/graphite anodes (2.2 mAh cm^{-2}) taken out of the multi-layer pouch-type full cells (3450 cell) after fast-charging cycling (6C charge/1C discharge). The cells were disassembled at fully charged states (SOC 100%) after 50 cycles.

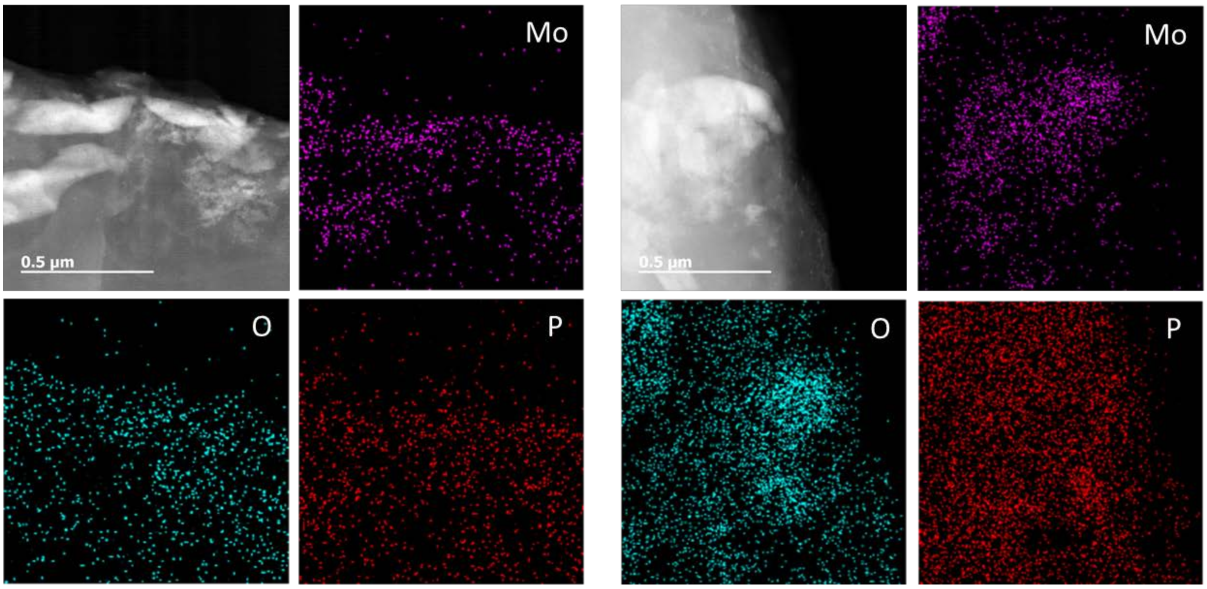

Supplementary Figure 18. TEM images and EDS mappings of the Mo-CP/graphite anode (2.2 mAh cm^{-2}) subjected to 300 cycles.

Supplementary Table 7. Material specifications of graphite received from Ningxia BOLT Technologies Co., Ltd., China).

Test items		Value
	d_{10} (μm)	7.30
Particle size distribution	d_{50} (μm)	17.51
	d_{90} (μm)	33.10
Moisture content (%)		0.15
Tap density (g cm^{-3})		1.03
Surface area (m^2 g^{-1})		2.20

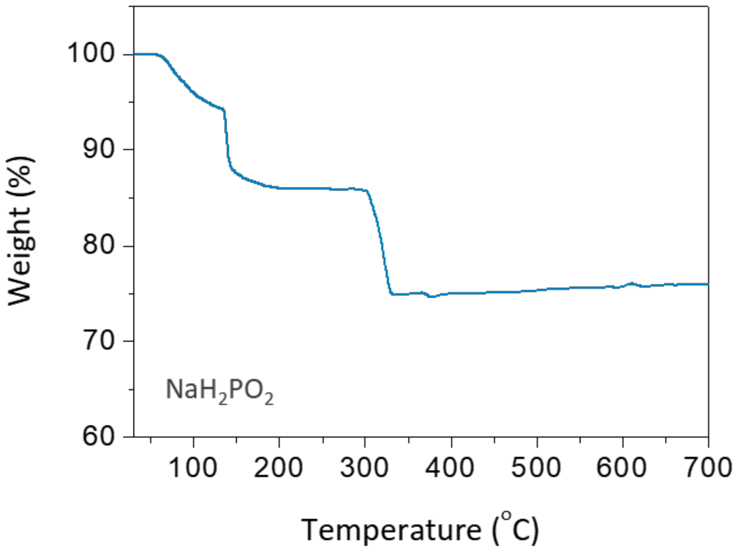

Supplementary Figure 19. Thermogravimetric analysis (TGA) of NaH_2PO_2 in N_2 with a heating rate of 3 °C min^{-1}. The TGA curve showed a significant weight loss at ~300 °C, which was attributed to the decomposition to PH_3 and H_2O gases[16,17].

Supplementary References

1. Wu, T., Pi, M., Zhang, D. & Chen, S. Three-dimensional porous structural MoP_2 nanoparticles as a novel and superior catalyst for electrochemical hydrogen evolution. *J. Power Sources* **328**, 551-557 (2016).
2. Kim, M. G., Lee, S. & Cho, J. Highly reversible Li-ion intercalating MoP_2 nanoparticle cluster anode for lithium rechargeable batteries. *J. Electrochem. Soc.* **156**, 89-94 (2009).
3. Landesfeind, J., Hattendorff, J., Ehrl, A., Wall, W. A. & Gasteiger, H. A. Tortuosity determination of battery electrodes and separators by impedance spectroscopy. *J. Electrochem. Soc.* **163**, A1373-A1387 (2016).
4. Kim, N., Chae, S., Ma, J., Ko, M. & Cho, J. Fast-charging high-energy lithium-ion batteries via implantation of amorphous silicon nanolayer in edge-plane activated graphite anodes. *Nat. Commun.* **8**, 812 (2017).
5. Cheng, Q., Yuge, R., Nakahara, K., Tamura N. & Miyamoto, S. KOH etched graphite for fast chargeable lithium-ion batteries. *J. Power Sources* **284**, 258-263 (2015).
6. Yuge, R., Tamura, N., Manako, T., Nakanp, K. & Nakahara, K. High-rate charge/discharge properties of Li-ion battery using carbon-coated composites of graphites, vapor grown carbon fibers, and carbon nanohorns. *J. Power Sources* **266**, 471-474 (2014).
7. Billaud, J., Bouville, F., Magrini, T., Villevieille, C. & Studart, A. R. Magnetically aligned graphite electrodes for high-rate performance Li-ion batteries. *Nat. Energy* **1**, 16097 (2016).
8. Cheng, Q. et al. Graphene-like-graphite as fast-chargeable and high-capacity anode materials for lithium ion batteries. *Sci. Rep.* **7**, 14782 (2017).
9. Qin, J. et al. Sandwiched C@SnO_2@C hollow nanostructures as an ultralong-lifespan high-rate anode material for lithium-ion and sodium-ion batteries. *J. Mater. Chem. A* **5**, 10946-10956 (2017).
10. An, W. et al. Dual carbon layer hybridized mesoporous tin hollow spheres for fast-rechargeable and highly-stable lithium-ion battery anodes. *J. Mater. Chem. A* **5**, 14422-14429 (2017).
11. Griffith, K. J., Wiaderek, K. M., Cibin, G., Marbella, L. E. & Grey, C. P. Niobium tungsten oxides for high-rate lithium-ion energy storage. *Nature* **559**, 556-563 (2018).
12. Lou, S. et al. Superior performance of ordered macroporous $TiNb_2O_7$ anodes for lithium ion batteries: Understanding from the structural and pseudocapacitive insights on achieving high rate capability. *Nano Energy* **34**, 15-25 (2017).
13. Shen, L., Chen, S., Maier, J. & Yu, Y. Carbon-coated Li_3VO_4 spheres as constituents of an advanced anode material for high-rate long-life lithium-ion batteries. *Adv. Mater.* **29**, 1701571 (2017).
14. Chen, Y. et al. A general strategy towards carbon nanosheets from triblock polymers as high-rate anode materials for lithium and sodium ion batteries. *J. Mater. Chem. A* **5**, 19866-19874 (2017).
15. Cheng, Q. & Zhang, Y. Multi-channel graphite for high-rate lithium ion battery. *J. Electrochem. Soc.* **165**, 1104-1109 (2018).
16. Song, L. & Zhang, S. A versatile route to synthesizing bulk and supported nickel phosphides by thermal treatment of a mechanical mixing of nickel chloride and sodium hypophosphite. *Powder Tech.* **208**, 713-716 (2011).
17. Li, P. & Zeng, H. C. Bimetallic Ni–Fe phosphide nanocomposites with a controlled architecture and composition enabling highly efficient electrochemical water oxidation. *J. Mater. Chem. A* **6**, 2231-2238 (2018).
